# Editorial: Combining computational and experimental approaches to characterize ion channels and transporters

**DOI:** 10.3389/fphys.2023.1320336

**Published:** 2023-11-08

**Authors:** Christoph Fahlke, Jan-Philipp Machtens, Walter Sandtner, Klaus Schicker, Han Sun

**Affiliations:** ^1^ Institute of Biological Information Processing, Molekular- und Zellphysiologie (IBI-1), Forschungszentrum Jülich, Jülich, Germany; ^2^ Center of Physiology and Pharmacology, Medical University of Vienna, Vienna, Austria; ^3^ Leibniz-Forschungsinstitut für Molekulare Pharmakologie (FMP), Berlin, Germany; ^4^ Institute of Chemistry, Technical University of Berlin, Berlin, Germany

**Keywords:** ion channels, ion transporter, computational biology, kinetic modeling, molecular dynamics simulation

Electrical signaling plays a major role in a variety of cellular functions. It is fast, well suited for coding information and is—because of the low dielectric constant of biological membranes—very energy-efficient. In living systems, ion channels and transporters determine electrical signals in a cooperative fashion. Transporters generate ion gradients, and ion channels utilize these gradients by mediating large ion fluxes that quickly charge the membrane capacitor and change voltages across the membrane. The importance of ion channels in excitable tissues has been known for many decades. The invention of patch clamping made a broad variety of cells accessible to cell electrophysiology; such experiments demonstrated the importance of electrical signaling also in many non-excitable cells over the human body.

Because of their widespread importance, ion channels and transporters have been intensively studied in the recent past. Currently, we have reached a solid understanding of the function of many ion channels and transporters, and we possess knowledge of the three-dimensional structures for most of them. Simultaneously, computational biology tools now enable us to link structure dynamics to transport functions. This rapid progress in understanding these class of membrane proteins prepares us for the next step, to use channel and transport function to predict ion concentrations and membrane potentials in cell organelles and cell compartments, towards an atomistic understanding of cell function.

To link channel and transporter structure to protein function and even more to the behavior of subcellular and cellular systems, experiments need to be combined with computational approaches. Our Research Topic *Combining Computational and Experimental Approaches to Characterize Ion Channels and Transporters*, has the aim to present recent progress and findings achieved through the application of such a combination. It encompasses eight publications that combine experiments and computational work to describe the function of various ion channels and transporters.

The mini-review by Hendriks et al. summarizes recent insights into the cation selectivity in ion channels. They describe multiple structures with distinct selectivity filter conformations that were discovered recently using a combination of structural biology, spectroscopic, and computational methods. Yüksel et al. combine atomistic MD simulation and electrophysiology for a better understanding of channel gating in HCN channels. They uncouple a linker region from the channel core by inserting one to five glycine residues. These modifications already abolish modulation by cAMP upon insertion of one glycine. In addition they accelerate activation kinetics. Simulations demonstrate increased mobility of the linker and the cyclic nucleotide-binding domains (CNBD) as well as destabilization of the channel gate. In HCN channels, hyperpolarizing voltage and cAMP binding are integrated by a mechanical continuum between the voltage sensor and the CNBD. This allows for the transmitting information to the periphery of the channel, thereby bypassing the pore. Gabriel et al. study ion binding within the selectivity filter of K^+^ channels using high bandwidth single channel recording and the blocker tetrapropylammonium. The authors determine voltage-dependent ion occupation probabilities by using a kinetic model of single-channel currents recorded in the absence of the blocker and extract release rate constants of the blocker from these data.

Five publications address transporters, and all of them describe transporter function with the help of kinetic models. Shi et al. focus on transporters that accumulate neutral and cationic amino acids in a Na^+^ and Cl^−^ dependent manner. Their analysis shows that the substrate turnover rate of this transporter is faster than for other SLC6 transporters, including the closely related glycine transporters. Whereas substrate translocation is fast (i.e., in sub-millisecond range), Na^+^ binding to the empty transporter is slow and possibly the rate-limiting partial reaction.

Walter Sandtner’s group explored how substrate concentrations on extra- and intracellular membrane sides can exert evolutionary pressure on the operating mode of a transporter (Schicker et al.). Their approach uses microscopic rate constants, which parameterize the kinetic models of solute carriers, to describe transporter function by analytical descriptors such as K_M_ and V_max_ for substrate transport. They hypothesize that the outcome of evolutionary adaptation must maximize substrate uptake rate at prevailing conditions and use an optimization algorithm to search for the microscopic rate constants, which yielded the largest possible value for the substrate uptake rate. This approach provided novel insights into how evolution may have shaped solute carrier function. In another study, the same group explored the general properties of allosteric modulation of solute carriers (Boytsov et al.). For this purpose, they use transition state theory and linear free energy relationships (LFER) to provide a theoretical framework for allosteric solute carrier modulation. Suslova et al. combine fast substrate application and kinetic modeling to understand the kinetic basis of gain-of-anion channel changes of the glial glutamate transporter EAAT1 in the pathomechanism in episodic ataxia (Winter et al., 2012; Kovermann et al., 2020). In this work, they also establish a method for statistical testing of fit parameters in kinetic transporter modeling. Barta et al. analyze how passive movements of transport substrates and ions affect secondary active transport. For this, they use a continuum mathematical model of liposomes with and without the sodium glucose transporter 1 (SGLT1) to assign water conductive states to the transport cycle and to improve the interpretation of substrate accumulation data in liposome systems.

Our Research Topic illustrates the potential of combining experiments and computational approaches. It shows how experiments can verify the results of molecular simulations and inspire new simulation studies. In addition, it demonstrates the usefulness of kinetic modeling for describing the mechanistic basis of secondary active transport and for discovering general principles of their function. Lastly, it provides an example how computational approaches that describe more complex experimental systems, i.e. liposomes, can significantly improve the interpretability of the obtained results. We are confident that computational approaches will be increasingly employed in membrane physiology and biophysics, and that we will see many contributions with a similar set of methods in the near future.
